# P-2099. Characterizing Substance Use in Older Adults Living with HIV

**DOI:** 10.1093/ofid/ofaf695.2263

**Published:** 2026-01-11

**Authors:** Shu Zhang, Charlene Thomas, Marshall J Glesby, Eugenia Siegler, Carrie Johnston

**Affiliations:** NewYork-Presbyterian Hospital - Weill Cornell, New York, NY; Weill Cornell Medicine, New York, New York; Weill Cornell Medicine, New York, New York; Division of Geriatrics and Palliative Medicine, Weill Cornell Medicine, New York, NY; Weill Cornell, New York, New York

## Abstract

**Background:**

Substance use in older people living with HIV (PWH) is understudied yet is of high clinical importance in relation to healthy aging. Prior data have suggested that rates of substance use disorder do not decline substantially with age in PWH, unlike in HIV- older adults, but further research is needed on how substance use patterns evolve with aging in PWH.

**Table 1.**
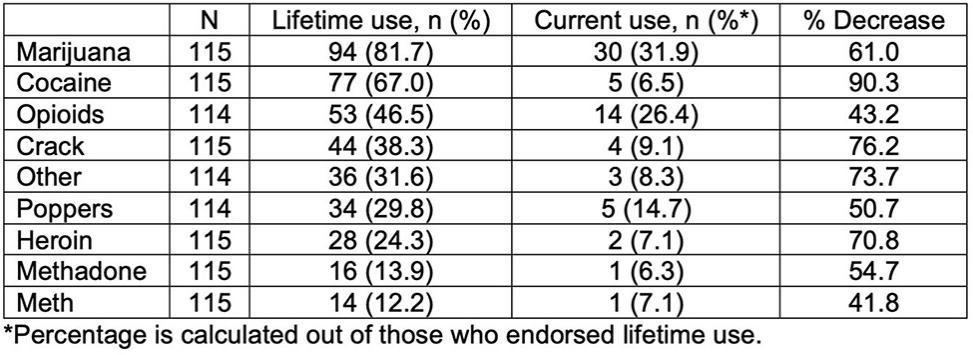
Lifetime and current substance use, and percent decrease between the two.

**Figure 1.**
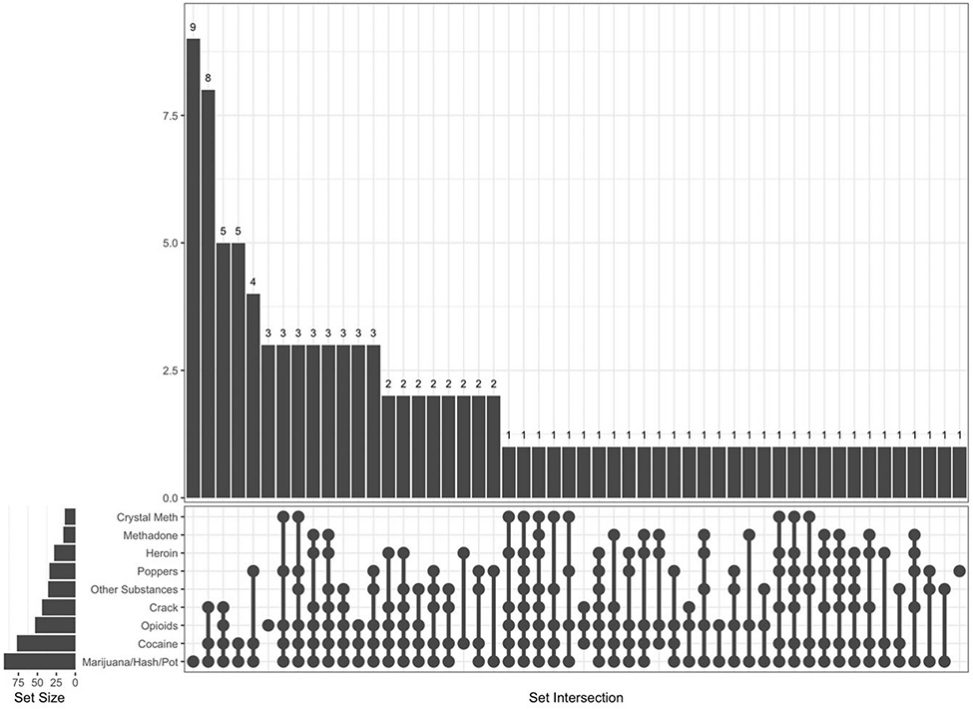
UpSet plot showing patterns of lifetime substance use.

**Methods:**

We conducted a sociodemographic survey as part of a longitudinal cohort study of older PWH at an urban academic medical center in New York City. Study participants were asked about tobacco use as current, former, or never use, and alcohol use was assessed using the AUDIT-C scale. Participants were also asked about lifetime use of substances of addictive potential, and if lifetime use was endorsed, they were asked about use within the prior 3 months (current use).

**Figure 2.**
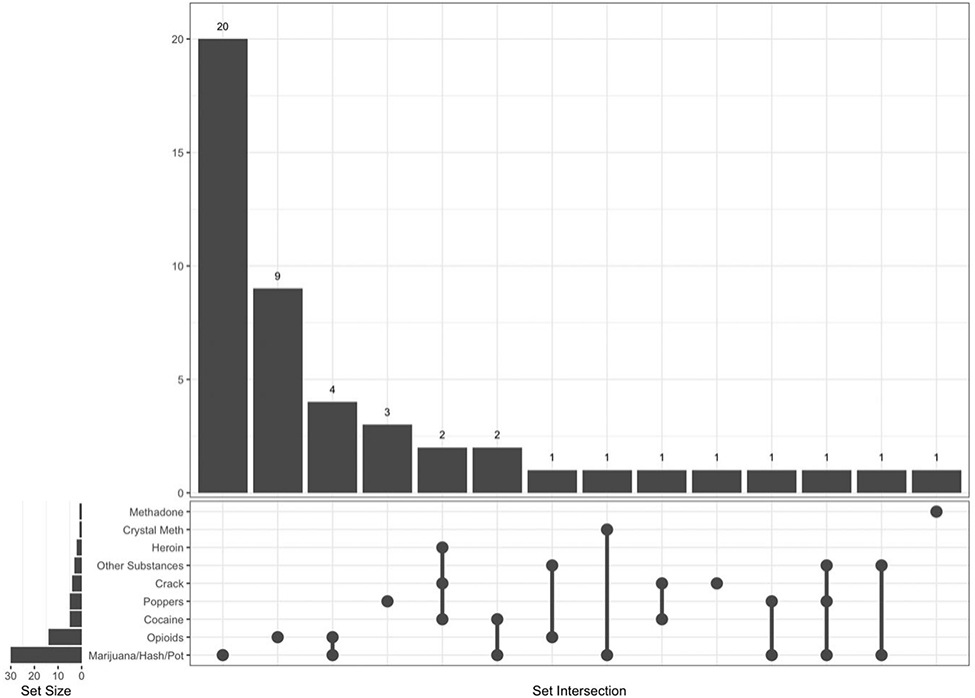
UpSet plot showing patterns of current substance use.

**Results:**

Overall, 115 adults with HIV completed the in-person study visit between 2022-2024. The group was 63% male and 50% Black, with a median age of 65 (range 56-82). The median AUDIT-C score was 1, for both male and female participants. When asked how often they had six or more drinks on one occasion in the last year, 40% responded never, and 8% responded with the maximum option of four or more times per week. Out of 91 individuals who responded regarding tobacco use, 52% were former smokers and 20% were current smokers. The most common drugs for lifetime use were marijuana (82%), cocaine (67%), and opioids (46%) (Table 1). For current use, marijuana remained the most prevalent (32%), with opioids following (26%), the majority of which were prescribed. Participants with lifetime use of marijuana also tended to have used other substances (Figure 1), but current use showed a shift toward isolated marijuana use (Figure 2). There was a substantial decline between lifetime use and current use for all drugs, with the greatest relative decrease in cocaine and crack.

**Conclusion:**

Comparisons between lifetime and current use of substances in this group suggest an overall decrease in substance use with aging. The motivations underlying decreased versus continued substance use warrant further study to facilitate caring for PWH as this group ages. Future directions will also include examining the relationship between substance use and quality of life indicators such as pain.

**Disclosures:**

All Authors: No reported disclosures

